# The influence of cervical spine rehabilitation on bioelectrical activity (sEMG) of cervical and masticatory system muscles

**DOI:** 10.1371/journal.pone.0250746

**Published:** 2021-04-26

**Authors:** Renata Kielnar, Anna Mika, Dorota Bylina, Jarosław Sołtan, Artur Stolarczyk, Błażej Pruszczyński, Henryk Racheniuk, Jan Szczegielniak, Aleksandra Królikowka, Łukasz Oleksy

**Affiliations:** 1 Institute of Health Sciences, Medical College of Rzeszow University, Rzeszów, Poland; 2 Institute of Clinical Rehabilitation, University of Physical Education in Krakow, Krakow, Poland; 3 Chair of Natural Sciences, Faculty of Physical Education in Biala Podlaska, The Josef Pilsudski University of Physical Education in Warsaw, Warsaw, Poland; 4 Foreign Languages Department, Faculty of Physical Education in Biala Podlaska, The Josef Pilsudski University of Physical Education in Warsaw, Warsaw, Poland; 5 Orthopaedic and Rehabilitation Department, Medical University of Warsaw, Warsaw, Poland; 6 Department of Orthopaedics and Paediatric Orthopaedics Medical University of Lodz, Lodz, Poland; 7 Institute of Physiotherapy, Faculty of Physical Education and Physiotherapy, Opole University of Technology, Opole, Poland; 8 Division of Sports Medicine, Department of Physiotherapy, Faculty of Health Sciences, Wroclaw Medical University, Wrocław, Poland; 9 Oleksy Medical & Sports Sciences, Lancut, Poland; Prince Sattam Bin Abdulaziz University, College of Applied Medical Sciences, SAUDI ARABIA

## Abstract

**Background:**

Coexistence of temporomandibular joint discomfort along with cervical spine disorders is quite common, and is associated with many limitations and adverse symptoms for the patient. Both diagnostics and treatment of these ailments are difficult, and in many cases, the effects of therapy are not satisfactory. This study assessed the impact of a 3-week neck-only rehabilitation programme without direct intervention in the craniofacial area on the bioelectric activity of both the cervical spine and muscles in the craniofacial area among patients with idiopathic neck pain who do not report TMJ pain.

**Design:**

A parallel group trial with follow-up; Setting: Rehabilitation Clinic.

**Methods:**

Twenty five patients experiencing idiopathic neck pain underwent the 3-week rehabilitation programme. Thirty five age-matched subjects with no cervical spine and temporomandibular joint (TMJ) dysfunctions were control group. At baseline and after 3 weeks the cervical and craniofacial area muscles’ bioelectrical activity (sEMG) was evaluated.

**Results:**

In the experimental group during cervical flexion, a significant decrease of sEMG amplitude was noted in the right (mean 25.1 μV; 95% CI: 21.5–28.6 vs mean 16.8 μV; 95% CI: 13.8–19.7) and left (mean 25.9 μV; 95% CI: 21.7–30.0 vs mean 17.2 μV; 95% CI: 13.6–20.7) Sternocleidomastoid as well as a significant increase in sEMG amplitude of the right (mean 11.1 μV; 95% CI: 7.9–14.2 vs mean 15.7 μV; 95% CI: 12.1–19.2) and left (mean 15.3 μV; 95% CI: 11.9–18.6 vs mean 20.2 μV; 95% CI: 15.7–24.2) Upper Trapezius muscles. In the experimental group, after therapy right and left Sternocleidomastoid, Temporalis Anterior and Masseter muscles presented lower fatigue levels.

**Conclusions:**

Three weeks of rehabilitation without any therapeutic intervention in temporomandibular joint significantly decreased the bioelectrical activity of the neck and craniofacial muscles while improving the muscle pattern of coactivation in participants with idiopathic neck pain who do not report temporomandibular joint pain. These observations could be helpful in the physiotherapeutic treatment of neck and craniofacial area dysfunctions.

**Trial registration:**

ID ISRCTN14511735—retrospectively registered.

## Introduction

Cervical spine dysfunction occurs in about 70% of the population [1]. Both diagnostics and treatment of these ailments are difficult, and in many cases, the effects of therapy are not satisfactory [2]. Therefore, a lot of researchers and clinicians are looking for new methods of neck pain evaluation and effective treatment. This research concerns the relationship between the neck and the masticatory system [3–7]. Coexistence of temporomandibular joint discomfort along with cervical spine disorders is quite common, and is associated with many limitations and adverse symptoms for the patient [4, 5]. There are studies describing the relationship between body posture and temporomandibular dysfunctions (TMDs) [8–10]. Researchers have suggested that if proprioceptive information from the masticatory system muscles is not accurate, it may disrupt the control of head and body position [11–13].

There are some reports describing the occurrence of disorders in the cervical spine of patients with diagnosed temporomandibular joint dysfunctions, however, the exact relationship between neck ailments and TMDs is still unclear [12–14]. Some authors have reported a relationship between craniofacial and neck pain, including biomechanical, neuroanatomical and neurophysiological aspects [15, 16]. Incorrect tension of the masticatory muscles was found to be associated with head posture and was suggested as one of the causes of dysfunctions in cervical paravertebral muscles [12, 17]. The possible explanation could be the neurophysiologic connections between the cervical spine and temporomandibular area, such as the convergence of trigeminal and upper cervical afferent inputs in the trigeminocervical nucleus [16]. A lot of controversy related to both the diagnosis of this complex problem and its treatment means that the therapy usually focuses on treating either the disorders in the temporomandibular joint alone, or only complaints regarding the cervical spine without a comprehensive approach to the problem [6, 11, 18]. Therefore, there is still a lack of studies reporting the clinically important results of such therapy. Nowadays, there are many studies using objective methods of muscle function evaluation. One of them is surface electromyography (sEMG), which may comprehensively assesse bioelectrical muscle activity, measuring their fatigue or neuromuscular coordination [4, 19–21].

The aim of this study was to assess the impact of a 3-week neck-only rehabilitation programme without direct intervention in the craniofacial area on the bioelectric activity of both the cervical spine and muscles in the craniofacial area among patients with idiopathic neck pain who do not report TMJ pain. The novelty of this study was verification of the hypothesis stating that rehabilitation focused on structures lying within the cervical spine can also affect the tension of the muscles of the temporomandibular joints. Our hypothesis assumes that therapy focusing only on the cervical region may have real impact on the structures located in the craniofacial area reducing muscular imbalance, and thus, it has a broader effect than previously thought. Based on the neurophysiological and biomechanical relationships between TMJ and the neck, it may be hypothesised that patients with idiopathic neck pain may also experience some TMJ dysfunctions, even those asymptomatic.

## Material and methods

### Participants

**Fig 1:**
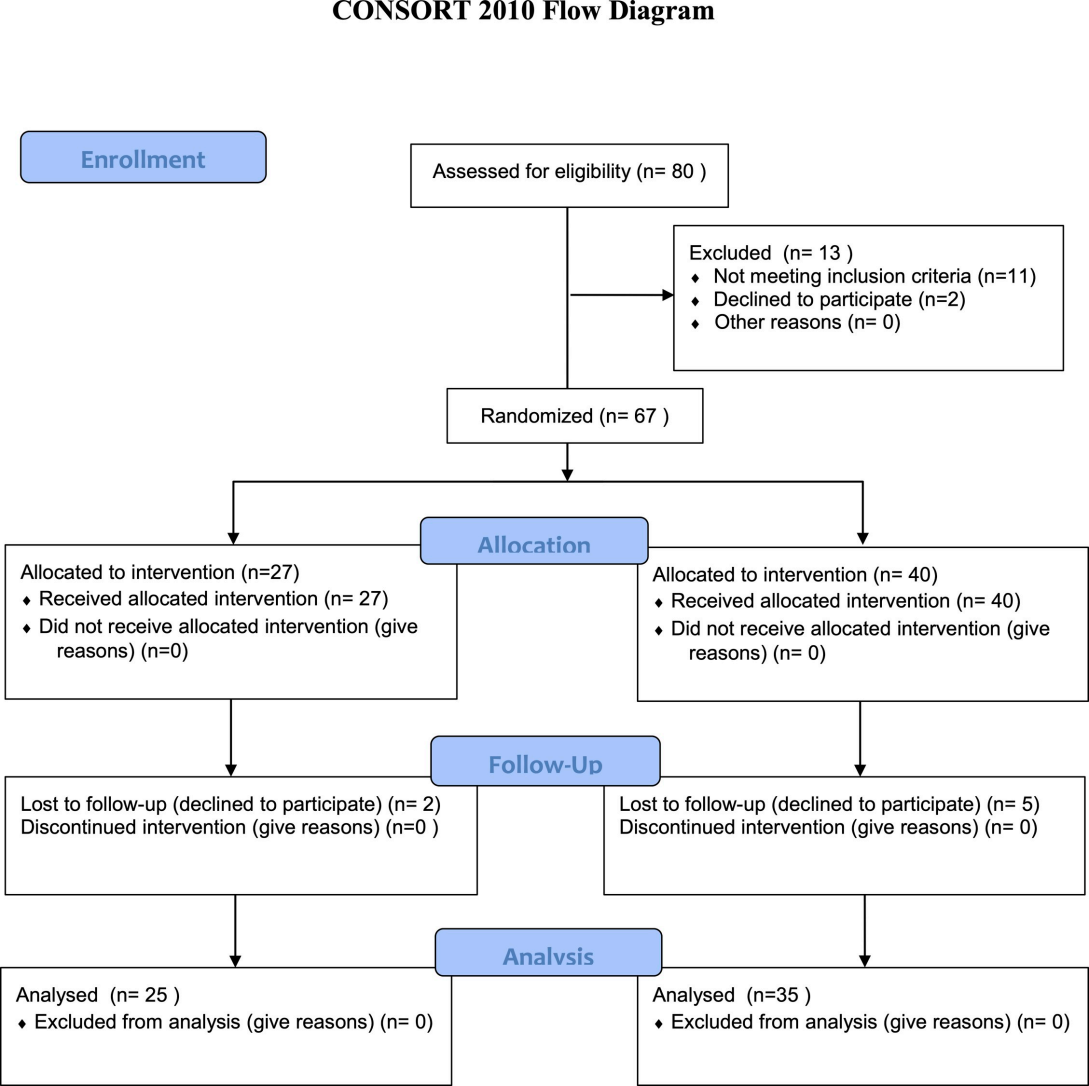
Consort flow diagram.

The study included 60 participants divided into 2 groups (Table 1, Fig 1). All subjects were allocated to an experimental (with neck pain) or control (pain -free) group based on the physician’s assessment who confirmed the diagnosis of idiopathic cervical pain. All subjects from the experimental group were patients of the Rehabilitation Clinic who received ambulatory treatment. They were recruited from patients admitted to the Clinic. Age matched control subjects were recruited from the local community.

**Table 1:** Patients’ characteristics.

	Experimental Group	Control Group
Number of subjects (n)	25	35
Sex	21 women, 4 men	29 women, 6 men
Age (years)	27–57 (38.5±8.52)	27–47 (35.1±5.65)
Body mass (kg)	62±11.9	64.3±14.1
Body height (cm)	164.6±6.3	166.2±5.3
Ethnicity	Caucasian	Caucasian

Group 1 (experimental)—n = 25, experiencing idiopathic neck pain and underwent the 3-week rehabilitation programme.

Exclusion criteria: cervical spine injury 3 months prior to the therapy; regular use of painkillers or steroids, without possibility to withdraw their usage for the whole duration of the therapy; radiographically diagnosed developmental and degenerative abnormalities of the cervical spine such as; orthodontic treatment (braces, aligners); removable dentures.

Group 2 (control)—n = 35, cervical pain free, with no cervical spine and temporomandibular joint (TMJ) dysfunctions or were not in the process of current orthodontic treatment.

All participants were informed in detail about the research protocol and gave their written informed consent to participate in the study. This study was approved by Ethical Committee of Rzeszow University in Poland. All procedures were performed in accordance with the 1964 Helsinki declaration and its later amendments. This study was registered in the ISRCTN registry. Registration number: ID ISRCTN14511735.

### Experimental procedures

**Fig 2:**
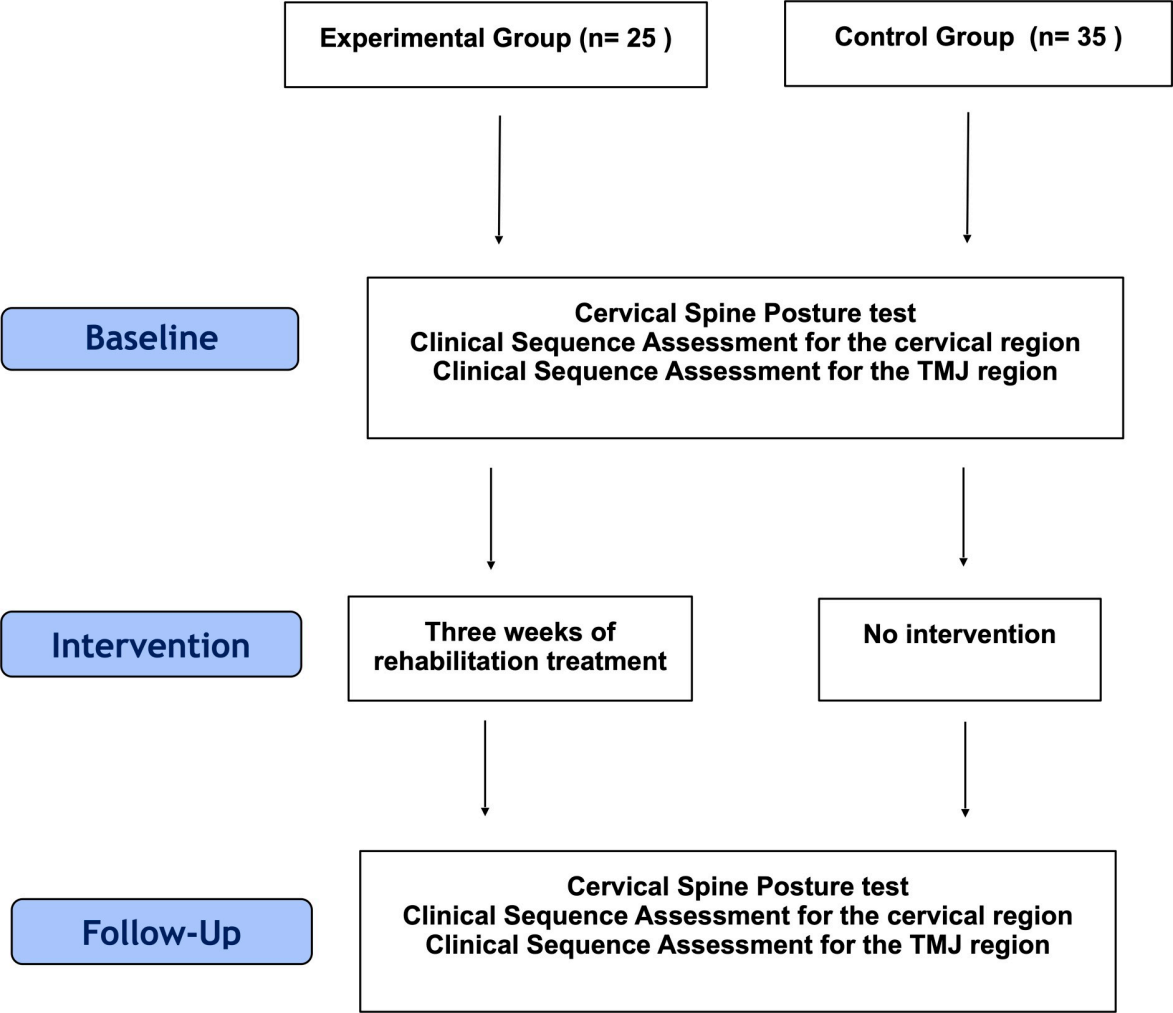
Flow diagram of the study procedure.

All measurements were performed twice, at baseline and after 3 weeks of treatment in the experimental group, and with a 3-week time interval in the control group (Fig 2).

### Therapeutic interventions

The experimental group underwent a 3-week rehabilitation programme, which was individual for each patient and comprised the following standard treatments for chronic pain of the musculoskeletal system: [22–24].

- manual therapy (soft tissue therapy of the neck and the shoulder girdle, trigger point therapy, manual cervical traction, classical massage, myofascial release;

The techniques were performed for 30 minutes each on the soft tissues which required relaxation depending on the needs of the therapy at a given time, the patient’s well-being and progress of the whole therapy. Each time, manual cervical traction was performed in lying position at the end of manual soft tissue therapy.

- individual exercises with a therapist (active exercise, respiratory re-education, body posture correction exercises);

Respirator re-education included 3 series of 5–7 active breaths. Active exercise comprised active correction of body posture, activation and training of the deep neck flexors, neck extensors and muscles stabilizing and rotating the shoulder girdle. Each time, the exercises were performed with a therapist in 3 series of 10 repetitions. The progression included an increase of repetitions, change of base position or increase in external resistance.

- Physical therapy (sollux lamp);

Applied each time before the massage after soft tissue therapy, 15 minutes with a blue filter on the shoulder girdle and cervical spine.

- Education on the nature of the dysfunction, body posture correction techniques, sleep and everyday-life ergonomics;

Rehabilitation was carried out 5 times a week, lasting about 2 hours per session. Participants from the control group did not undergo any therapy.

### sEMG measurements

Bioelectrical muscle activity was recorded in accordance with SENIAM guidelines [25]. Prior to electrode placement, the skin was cleaned with alcohol. Surface electrodes (Ag/AgCl) (Sorimex, Poland) with a 2 cm centre-to-centre distance were attached along the direction of the muscle fibres on the muscle bellies. The signals were registered with a 16-bit accuracy at a sampling rate of 1,500 Hz using the *Noraxon G2 TeleMyo 2400* unit (Noraxon USA, Inc., Scottsdale, AZ). The sEMG signal was processed using the *MyoResearch XP* software (Noraxon USA, Inc.,Scottsdale, AZ) [25, 26].

sEMG data was filtered using the built-in hardware 1^st^ order high-pass filter set to 10 Hz +/- 10% cut-off. The raw sEMG data were visually checked for artefacts. The sEMG signal was rectified and then the root mean squared (RMS) value was determined over a 200-msec window [26].

The muscles’ bioelectrical activity was evaluated during 3 tests: Cervical Spine Posture test, Clinical Sequence Assessments for Cervical region and Clinical Sequence Assessments for the TMJ region [27, 28]. All tests were performed by an experienced researcher, who was blinded to the subject group allocation.

### Cervical Spine Posture test

The muscle bioelectrical activity (sEMG) was measured while seated, with corrected head posture (the subject was asked by researcher to assume a position with the head within body axis avoiding protracted head position).

The following muscles were evaluated:

- Right and Left Sternocleidomastoid (SCM);
- Right and Left Temporalis Anterior (TA);
- Right and Left Masseter (MASS);
- Right and left Cervical Paraspinals (C4 region) (CERV).

The Cervical Spine Posture test consisted of 3 activities:

1. Baseline/Postural Evaluation (10-second rest)—the subject was instructed to sit comfortably with the muscles at rest.
2. Functional Clench (two, 2-second maximal clenching of the teeth, with a 5-second resting interval in-between).
3. Functional Clench with Control (two, 2-second maximal clenching of the teeth with a cotton swab between the molar teeth, with a 5-second resting interval in-between).

For each activity of the Cervical Spine Posture test, the Average Mean Amplitude (uV) of the sEMG signal was calculated.

### Clinical Sequence Assessment for the cervical region

The bioelectrical muscle activity (sEMG) was measured while seated, with habitual head posture.

The following muscles were evaluated:

- Right and Left Sternocleidomastoid (SCM);
- Right and Left Upper Trapezius (UP TRA);
- Right and left Cervical Paraspinals (C4 region) (CERV).

Clinical Sequence Assessments for the Cervical region consisted of 4 activities:

1. Baseline/Postural Evaluation (10-second rest)—the subject was instructed to sit comfortably with the muscles at rest.
2. Cervical Flexion and Return to neutral (5-second cervical spine flexion, 2 seconds of rest in flexed position and 5-second return from flexion to neutral position).
3. Cervical Right and Left Rotation (5 seconds of cervical spine rotation to the right and 5-second return to neutral position, 2-second rest in neutral position and then 5-second cervical spine rotation to the left and 5-second return to neutral position).
4. Cervical Right and Left Lateral Flexion (5-second cervical spine lateral bending in coronal plane to the right and 5-second return to neutral position, 2 seconds of rest in neutral position and then 5-second cervical spine lateral bending in coronal plane to the left and 5-second return to neutral position).

For each activity of Clinical Sequence Assessments for the Cervical region, the Average Mean Amplitude (uV) of sEMG signal was calculated.

### Clinical Sequence Assessment for the TMJ region

The muscles’ bioelectrical activity (sEMG) was measured while seated, with habitual head posture.

The following muscles were evaluated:

- Right and Left Sternocleidomastoid (SCM);
- Right and Left Temporalis Anterior (TA);
- Right and Left Masseter (MASS);
- Right and left Cervical Paraspinals (C4 region) (CERV).

Clinical Sequence Assessments for TMJ region consisted of 4 activities:

1. Baseline/Postural Evaluation (10-second rest)—the subject was instructed to sit comfortably with the muscles at rest.
2. Functional Clench (two, 2-second maximal clenching of the teeth, with a 5–second interval for rest in-between).
3. Functional Clench with Control (two, 2-second maximal clenching of the teeth with a cotton swab between the molar teeth, with a 5-second resting interval in-between).
4. Prolonged Clench with Control—Fatigue Analysis (one, 10-second maximal clenching of the teeth with a cotton swab between the molar teeth).

For the 1^st^, 2^nd^ and 3^th^ activity of Clinical Sequence Assessments for the TMJ region, the Average Mean Amplitude (uV) of sEMG signal was calculated. For the 4^th^ activity, Change of Mean Amplitude (uV) of the sEMG signal was calculated.

### Statistical analysis

Statistical analysis was carried out using the STATISTICA 12.0 software. To assess the normality of variable distribution, the Shapiro-Wilk test was performed. Two-way ANOVA was performed to determine the significance of differences in the evaluated variables, where one of the main factors was the comparison between groups (group: experimental vs. control), and the other main factor was the repeated measure (time: baseline vs. 3 weeks). Then, Tukey’s post-hoc test was performed. The effect size was calculated using Cohen’s *d*. Differences were considered to be statistically significant at the assumed level of significance (*p*<0.05). Paired t-test power analysis of rehabilitation influence determined that at least 25 subjects were required to obtain a power of 0.8 at a two-sided level of 0.05 with the effect size of d = 0.8.

## Results

### Cervical Spine Posture test

After the therapy in the experimental group, all evaluated muscles were less active during the same tasks compared to their pre-therapy (baseline) activity. During resting activity, we observed a significant decrease of sEMG amplitude in the right and left SCM muscles compared to baseline. At rest, there were no significant changes in TA, MASS or CERV muscles, similarly as in control group (*p*>0.05) (Table 2). In the experimental group during functional clench, a significantly lower sEMG amplitude was noted in the right and left SCM muscles, in the left TA muscle and in the right MASS muscle compared to pre-therapy values. There were no significant changes in CERV muscles (*p*>0.05) (Table 3). During functional clench with a control in the experimental group, a significant decrease was noted in sEMG amplitude in the left TA and right and left MASS muscles. In the control group, there were no significant changes (*p*>0.05) (Table 4).

**Table 2:** Changes in muscles bioelectrical activity during Cervical Spine Posture test at Baseline/Postural Evaluation (10-second rest) phase.

Outcome Measure		Experimental Group	p#	ES#	Control Group	p#	ES#	p*	p**
SCM L (μV)	Baseline	7.8±5.1 (5.6–9.9)	**0.008**	**0.54**	3.7±1.9 (3.0–4.3)	n.s.	0.05	**0.01**	n.s.
	Post	5.5±3.1 (4.2–6.7)			3.6±1.5 (3.0–4.1)			n.s.	
SCM R (μV)	Baseline	7.2±4.7 (5.2–9.1)	**0.006**	**0.50**	4.2±2.2 (3.4–4.9)	n.s.	0.05	**0.01**	n.s.
	Post	5.2±3.0 (3.9–6.4)			4.3±1.3 (3.8–4.7)			n.s.	
TA L (μV)	Baseline	7.8±5.8 (5.4–10.1)	n.s.	0.07	6.5±1.8 (5.8–7.1)	n.s.	0.04	n.s.	n.s.
	Post	7.4±4.3 (5.6–9.1)			6.4±2.8 (5.4–7.3)			n.s.	
TA R (μV)	Baseline	9.0±7.2 (6.0–11.9)	n.s.	0.40	6.9±2.0 (6.2–7.5)	n.s.	0.05	n.s.	n.s.
	Post	6.8±2.4 (5.8–7.7)			6.7±2.7 (5.7–7.6)			n.s.	
MASS L (μV)	Baseline	4.1±1.9 (3.3–4.8)	n.s.	0.07	3.5±1.6 (2.9–4.0)	n.s.	0.04	n.s.	n.s.
	Post	4.3±3.3 (2.9–5.6)			3.5±1.7 (2.9–4.0)			n.s.	
MASS R (μV)	Baseline	3.5±2.6 (2.4–4.5)	n.s.	0.45	3.3±1.4 (2.8–3.7)	n.s.	0.04	n.s.	n.s.
	Post	2.6±1.1 (2.1–3.0)			3.6±1.9 (2.9–4.2)			n.s.	
CERV L (μV)	Baseline	5.1±2.5 (4.0–6.1)	n.s.	0.16	4.3±1.3 (3.8–4.7)	n.s.	0.02	n.s.	n.s.
	Post	5.6±3.6 (4.1–7.0)			4.4±1.3 (3.9–4.8)			n.s.	
CERV R (μV)	Baseline	4.6±1.7 (3.8–5.3)	n.s.	0.05	3.5±1.3 (3.0–3.9)	n.s.	0.04	n.s.	n.s.
	Post	4.7±2.0 (3.8–5.5)			3.6±1.3 (3.1–4.0)			n.s.	

p#—p value between baseline and post- therapy within each group (time main effect).

p*—p value between study groups (group main effect).

ES#–effect size (Cohen d) within each group.

p**–p value of main effects interaction.

Values are expressed as Mean ± SD (95% Confidence Interval).

**Table 3:** Changes in muscles bioelectrical activity during Functional Clench phase.

Outcome Measure		Experimental Group	p#	ES#	Control Group	p#	ES#	p*	p**
SCM L (μV)	Baseline	7.4±4.3 (5.6–9.1)	**0.001**	**0.92**	5.1±2.4 (4.2–5.9)	n.s.	0.04	**0.01**	n.s.
	Post	4.0±2.9 (2.8–5.1)			5.0±2.2 (4.2–5.7)			n.s.	
SCM R (μV)	Baseline	6.9±2.8 (5.7–8.0)	**0.001**	**0.77**	5.7±2.2 (4.9–6.4)	n.s.	0.09	n.s.	n.s.
	Post	4.7±2.9 (5.5–5.8)			5.5±1.8 (4.8–6.1)			n.s.	
TA L (μV)	Baseline	29.1±7.6 (25.9–32.2)	**0.001**	**0.97**	28.9±8.8 (25.8–31.9)	n.s.	0.09	n.s.	n.s.
	Post	22.1±6.7 (19.3–24.8)			28.1±7.8 (25.4–30.7)			**0.01**	
TA R (μV)	Baseline	28.4±10.4 (24.1–32.6)	**0.003**	**0.55**	24.6±9.1 (21.4–27.7)	n.s.	0.01	n.s.	n.s.
	Post	23.2±8.1 (19.8–26.5)			24.5±7.9 (21.7–27.2)			n.s.	
MASS L (μV)	Baseline	27.0±7.8 (23.7–30.2)	n.s.	0.13	26.4±8.5 (23.4–29.3)	n.s.	0.03	n.s.	n.s.
	Post	25.8±9.3 (21.9–29.6)			26.1±7.5 (23.5–28.6)			n.s.	
MASS R (μV)	Baseline	24.4±9.1 (20.6–28.1)	**0.001**	**0.32**	24.1±6.2 (21.9–26.2)	n.s.	0.18	n.s.	n.s.
	Post	21.7±7.6 (18.5–24.8)			25.1±4.7 (23.4–26.7)			**0.04**	
CERV L (μV)	Baseline	5.1±2.9 (3.9–6.2)	n.s.	0.34	4.9±1.9 (4.2–5.5)	n.s.	0.10	n.s.	n.s.
	Post	5.9±1.6 (5.2–6.5)			4.7±2.0 (4.0–5.3)			n.s.	
CERV R (μV)	Baseline	4.4±1.7 (3.6–5.1)	n.s.	0.29	4.1±1.9 (3.4–4.7)	n.s.	0.11	n.s.	n.s.
	Post	5.0±2.3 (5.6–2.3)			4.3±1.7 (3.7–4.8)			n.s.	

p#—p value between baseline and post- therapy within each group (time main effect).

p*—p value between study groups (group main effect).

ES#–effect size (Cohen d) within each group.

p**–p value of main effects interaction.

Values are expressed as Mean ± SD (95% Confidence Interval).

**Table 4:** Changes in muscles bioelectrical activity during Functional Clench with Control phase.

Outcome Measure		Experimental Group	p#	ES#	Control Group	p#	ES#	p*	p**
SCM L (μV)	Baseline	7.9±4.1 (6.2–9.5)	n.s.	0.37	7.1±3.9 (5.7–8.4)	n.s.	0.05	n.s.	n.s.
	Post	6.6±2.6 (5.5–7.6)			6.9±3.4 (5.7–8.0)			n.s.	
SCM R (μV)	Baseline	7.6±3.6 (6.1–9.0)	n.s.	0.14	7.3±3.2 (6.2–8.3)	n.s.	0.02	n.s.	n.s.
	Post	7.1±3.1 (5.8–8.3)			7.2±3.6 (5.9–8.4)			n.s.	
TA L (μV)	Baseline	27.4±8.6 (23.8–30.9)	**0.001**	**0.51**	23.7±6.8 (21.3–26.0)	n.s.	0.09	**0.01**	n.s.
	Post	23.5±6.4 (20.8–26.1)			23.1±5.8 (21.1–25.0)			n.s.	
TA R (μV)	Baseline	25.9±9.6 (21.9–29.8)	n.s.	0.18	24.1±7.0 (21.6–26.5)	n.s.	0.07	n.s.	n.s.
	Post	24.2±8.6 (20.6–27.7)			23.6±6.9 (21.2–25.9)			n.s.	
MASS L (μV)	Baseline	29.5±6.8 (26.6–32.3)	**0.003**	**0.60**	27.2±7.5 (24.6–29,7)	n.s.	0.01	n.s.	n.s.
	Post	24.5±9.6 (20.5–28.4)			27.3±8.5 (24.3–30.2)			n.s.	
MASS R (μV)	Baseline	31.7±7.2 (28.7–34.6)	**0.001**	**0.96**	29.8±6.2 (27.6–31.9)	n.s.	0.24	n.s.	n.s.
	Post	24.6±7.5 (21.5–27.6)			28.3±6.1 (26.2–30.3)			**0.04**	
CERV L (μV)	Baseline	4.2±2.6 (3.1–5.2)	n.s.	0.22	4.9±1.9 (4.2–5.5)	n.s.	0.35	n.s.	n.s.
	Post	4.8±2.7 (3.6–5.9)			4.2±2.0 (3.5–4.8)			n.s.	
CERV R (μV)	Baseline	4.5±2.1 (3.6–5.3)	n.s.	0.16	4.3±1.7 (3.7–4.8)	n.s.	0.11	n.s.	n.s.
	Post	4.9±2.7 (3.7–6.0)			4.1±1.8 (3.4–4.7)			n.s.	

p#—p value between baseline and post- therapy within each group (time main effect).

p*—p value between study groups (group main effect).

ES#–effect size (Cohen d) within each group.

p**–p value of main effects interaction.

Values are expressed as Mean ± SD (95% Confidence Interval).

### Clinical Sequence Assessment for the cervical region

After therapy, in the experimental group during cervical flexion, a significant decrease of sEMG amplitude was noted in the right and left SCM as well as a significant increase in sEMG amplitude of the right and left UP TRA muscles. Lower sEMG amplitude was also observed in the right SCM muscle during return from the cervical flexion phase of the test (Table 5). There were no significant changes in CERV muscle activity or in the case of flexion during the return phases. Also, no changes were noted in all the evaluated muscles at rest, during cervical right and left rotation or cervical right and left lateral flexion (*p*>0.05). There were no significant changes in the control group (*p*>0.05).

**Table 5:** Changes in muscles bioelectrical activity during Clinical Sequence Assessments for the Cervical region at Cervical Flexion and Return to neutral phase.

Outcome Measure		Experimental Group	p#	ES#	Control Group	p#	ES#	p*	p**
SCM L (μV) flexion	Baseline	25.9±10.1 (21.7–30.0)	**0.003**	**0.92**	18.9±10.9 (15.1–22.6)	n.s.	0.02	n.s.	n.s.
	Post	17.2±8.6 (13.6–20.7)			18.7±8.8 (15.6–21.7)			n.s.	
SCM R (μV) flexion	Baseline	25.1±8.6 (21.5–28.6)	**0.003**	**1.05**	20.4±9.2 (17.2–23.5)	n.s.	0.09	n.s.	n.s.
	Post	16.8±7.1 (13.8–19.7)			19.9±7.6 (17.2–22.5)			n.s.	
UP TRA L (μV) flexion	Baseline	15.3±8.2 (11.9–18.6)	**0.001**	**0.50**	12.7±4.8 (11.0–14.3)	n.s.	0.01	**0.01**	n.s.
	Post	20.0±10.4 (15.7–24.2)			12.7±4.6 (11.1–14.2)			n.s.	
UP TRA R (μV) flexion	Baseline	11.1±7.6 (7.9–14.2)	**0.001**	**0.56**	11.5±3.0 (10.4–12.5)	n.s.	0.30	n.s.	n.s.
	Post	15.7±8.6 (12.1–19.2)			10.6±2.9 (9.6–11.5)			n.s.	
CERV L (μV) flexion	Baseline	11.6±7.1 (3.6–14.5)	n.s.	0.06	11.3±4.5 (9.7–12.8)	n.s.	0.12	n.s.	n.s.
	Post	11.1±7.6 (7.9–14.2)			10.8±3.5 (9.5–12.0)			n.s.	
CERV R (μV) flexion	Baseline	10.9±6.2 (8.3–13.4)	n.s.	0.14	10.4±4.2 (8.9–11.8)	n.s.	0.07	n.s.	n.s.
	Post	9.9±7.5 (6.8–12.9)			10.7±4.1 (9.2–12.1)			**0.04**	
SCM L (μV) return	Baseline	19.8±8.3 (16.3–23.2)	n.s.	0.38	16.2±5.9 (14.1–18.2)	n.s.	0.08	**0.02**	n.s.
	Post	17.0±6.3 (14.3–19.6)			15.7±6.0 (13.6–17.7)			n.s.	
SCM R (μV) return	Baseline	19.5±7.5 (16.4–22.5)	**0.003**	**0.71**	16.4±4.7 (14.7–18.0)	n.s.	0.18	**0.02**	n.s.
	Post	14.4±6.7 (11.6–17.1)			15.5±4.8 (13.8–17.1)			n.s.	
UP TRA L (μV) return	Baseline	14.2±5.4 (11.9–19.4)	n.s.	0.48	12.7±3.8 (11.3–14.0)	n.s.	0.02	n.s.	n.s.
	Post	16.8±5.3 (14.6–18.9)			12.6±4.8 (10.9–14.2)			n.s.	
UP TRA R (μV) return	Baseline	15.8±8.5 (12.2–19.3)	n.s.	0.47	11.3±3.3 (10.1–12.4)	n.s.	0.35	**0.02**	n.s.
	Post	12.5±4.9 (10.4–14.5)			12.4±2.8 (11.4–13.3)			n.s.	
CERV L (μV) return	Baseline	14.9±6.8 (12.0–17.7)	n.s.	0.07	13.5±6.1 (11.4–15.5)	n.s.	0.05	n.s.	n.s.
	Post	14.4±6.2 (11.8–16.9)			13.2±4.9 (11.5–14.8)			n.s.	
CERV R (μV) return	Baseline	14.4±4.8 (12.4–16.3)	n.s.	0.06	13.6±4.8 (11.9–15.2)	n.s.	0.25	n.s.	n.s.
	Post	14.7±3.9 (13.0–16.3)			12.5±3.6 (11.2–13.7)			n.s.	

p#—p value between baseline and post- therapy within each group (time main effect).

p*—p value between study groups (group main effect).

ES#–effect size (Cohen d) within each group.

p**–p value of main effects interaction.

Values are expressed as Mean ± SD (95% Confidence Interval).

### Clinical Sequence Assessment for the TMJ region

In the experimental group, after therapy, all evaluated muscles (SCM, TA and MASS) presented lower fatigue levels during the 10-second Prolonged Clench with the Control phase of the test. The Change of Mean Amplitude during isometric contraction was significantly lower after therapy compared to baseline indicating a lower level of muscle fatigue (Table 6). We did not observe any significant differences in all resting phases, during functional clench and functional clench with a control (*p*>0.05). There were no significant changes in the control group (*p*>0.05).

**Table 6:** Changes in muscles bioelectrical activity during Clinical Sequence Assessments for TMJ region at Prolonged Clench with Control—Fatigue Analysis phase.

Outcome Measure		Experimental Group	p#	ES#	Control Group	p#	ES#	p*	p**
SCM L (μV)	Baseline	84.0±53.1 (62.0–105.9)	**0.0001**	**1.19**	93.5±41.9 (79.1–107.8)	n.s.	0.21	n.s.	n.s.
	Post	36.7±17.3 (29.5–43.8)			85.1±36.2 (72.6–97.5)			**0.001**	
SCM R (μV)	Baseline	88.4±43.7 (70.3–106.4)	**0.0001**	**1.68**	89.6±32.7 (78.3–100.8)	n.s.	0.07	n.s.	n.s.
	Post	33.5±15.0 (27.3–39.6)			86.8±39.6 (73.1–100.4)			**0.001**	
TA L (μV)	Baseline	46.6±21.8 (37.6–55.5)	**0.0001**	**1.99**	44.5±22.8 (36.6–52.3)	n.s.	0.04		n.s.
	Post	13.7±8.3 (10.2–17.1)			45.5±17.8 (39.3–51.6)			**0.001**	
TA R (μV)	Baseline	54.2±23.2 (44.6–63.7)	**0.0001**	**2.13**	38.6±16.4 (32.9–44.2)	n.s.	0.08	n.s.	n.s.
	Post	16.4±9.4 (12.5–20.2)			37.2±16.4 (31.5–42.8)			**0.001**	
MASS L (μV)	Baseline	44.9±19.7 (36.7–53.0)	**0.01**	**1.59**	43.1±12.7 (38.7–47.4)	n.s.	0.35	n.s.	n.s.
	Post	20.6±8.7 (17.0–24.1)			48.3±16.6 (42.5–54.0)			**0.01**	
MASS R (μV)	Baseline	57.2±28.3 (45.5–68.8)	**0.01**	**1.53**	44.8±22.2 (37.1–52.4)	n.s.	0.06	n.s.	n.s.
	Post	24.6±10.2 (20.3–28.8)			46.2±18.7 (39.7–52.6)			**0.02**	

p#—p value between baseline and post- therapy within each group (time main effect).

p*—p value between study groups (group main effect).

ES#–effect size (Cohen d) within each group.

p**–p value of main effects interaction.

Values are expressed as Mean ± SD (95% Confidence Interval).

## Discussion

The most important observation from this study is that 3 weeks of rehabilitation without any therapeutic intervention in TMJ significantly decreased the bioelectrical activity of the neck and craniofacial muscles while improving the muscle pattern of coactivation in participants with idiopathic neck pain who did not report TMJ pain. After the therapy the values of muscles bioelectrical activity were similar to that noted at baseline in the healthy control group, which may be considered as a point of reference during the therapy in subjects with idiopathic neck pain. Our results may suggest that the improvement in neck muscle function has a positive effect also on the functioning of the masticatory system.

There are some studies which evaluated the functional relationship between the craniofacial and the neck regions during head movements, during chewing or teeth clenching [29–31]. In some studies the therapy was focused solely on the orofacial region [32, 33], or on both–the neck and TMJ areas [6, 34, 35]. Even if the therapy was applied only to the neck, the study included patients with TMJ pain [7, 34]. There is a lack of studies describing how the rehabilitation focused only on the cervical spine in patients with idiopathic neck pain only, and with no pain in the TMJ affecting the functioning of the masticatory system. O’Leary et al. [36] examined the sEMG activity of the masseter and scalene anterior muscles during cervical spine flexion and noted a significant increase in their activity with the simultaneous increase in neck pain intensity. In our research, after 3 weeks of rehabilitation, the activity of the SCM muscles during neck flexion decreased significantly indicating their functional improvement. It was also reported that people with neck pain demonstrated greater activation of neck muscles compared to people without pain, which may suggest a specific pattern of motor control for compensation and protection of painful muscles [36, 37]. In our research, during the cervical spine flexion, interaction of the UP TRA and SCM muscles was also noted. We have suggested that it is also a type of compensation associated with the neck pain caused by an imbalance between the neck flexor muscles. After rehabilitation, there was significant improvement in the coactivation of those muscles with a decrease of SCM activity and an increase of the UP TRA muscle activity. This may indicate a positive effect of therapy and obtaining a favorable pattern of muscle activation during cervical flexion.

Some authors have suggested a relationship between neck muscle imbalance and forward head posture [3, 7, 38, 39]. Observations by other authors indicate that changes in the head position may cause pain and dysfunction of the cervical spine and masticatory system [40]. Excessive forward head posture disrupts the mechanics of the cervical spine and may affect deep muscle tension [10, 41]. Forsberg et al. [42] investigated different length and tone of the neck muscles which may affect masticatory muscle activity. In studies, it has been reported that forward head posture may be associated with aggravated thoracic kyphosis, increased activity of the cervical spine muscles such as masseters, paravertebral and upper trapezius [43]. Cortese et al. [44] have noted that forward head posture is a risk factor of TMDs. There are some reports suggesting that increased activity of the temporalis anterior muscles is observed in people with forward head posture and with weakness of the deep neck muscles. This situation also adversely affects the activity of the masseter muscles because they compensate the function of weak deep neck muscles by increasing coactivation of the paravertebral muscles [43, 45]. Edmondston et al. [46], in healthy people, assessed the influence of corrected sitting position on the sEMG activity of cervical spine extensor muscles, and they did not observe any differences between the activity of these muscles in habitual and corrected position. In our study, we also analysed muscle activity in corrected sitting position, but both in subjects with neck pain and in healthy controls. The resting activity of the SCM muscles significantly decreased after 3 weeks of therapy reaching sEMG amplitude values noted in the control group. Moreover, in corrected sitting position during functional clench and during clench with a control, SCM, TA and MASS muscle activity was also significantly lower after treatment compared to baseline. This may indicate the beneficial effect of the applied therapy on the functioning muscles and suggest that after therapy, the patients were able to better control posture correction while sitting. In clinical practice, posture correction is used in the treatment of people with neck dysfunction or with shoulder pain [38, 47]. McLean et al. [47] evaluated the influence of a therapeutic posture correction programme on the activity of the cervical spine, shoulder girdle and the masticatory system muscles. They reported a tendency towards decrease in the activation level of all the evaluated muscles while sitting in the corrected position.

Increased masticatory muscle fatigue may be a symptom of the dysfunction, but the reason for increased muscles fatigability is not fully understood. There are also a few studies in which fatigue of the masticatory muscles in patients with cervical spine disorders was assessed [48]. It was shown that fatigue of the masticatory muscles in head forward posture was greater compared to the position with the head in corrected position [45]. Some authors have evaluated masticatory muscle fatigue in both healthy people and those with temporomandibular dysfunctions [21, 45, 48]. Sforza et al. [48], in 10 healthy people, examined the sEMG activity of the masseter and temporalis anterior muscle during teeth clench, and observed significant changes in the median frequency in both masseter muscles. Castroflorio et al. [21], during teeth clench, have assessed masseter and temporalis anterior muscle fatigue in 20 healthy people and in 18 individuals with TMDs. They found symptoms of the evaluated muscle fatigue in both groups, but the fatigue was higher in people with temporomanadibular dysfunction compared to the healthy controls [21]. Similar results were reported by Park et al. [49] who evaluated the masseter and temporalis anterior muscles in 19 subjects diagnosed with unilateral TMJ degeneration, but without masticatory muscle pain, and in 20 healthy controls. During prolonged teeth clench, the decrease in mean frequency was greater among subjects with TMJ degeneration and the muscles on the dysfunctional side exhibited significantly higher fatigue than in the healthy controls. Some authors have indicated that there is a great tendency to intensify fatigue of the masticatory muscles in patients with temporomandibular dysfunction in relation to healthy people [42, 50]. Those observations are in agreement with our own results stating that in the experimental group, masticatory system muscle fatigue was higher in patients with idiopathic neck pain compared to controls, and this level decreased significantly after 3 weeks of therapy., The smaller change in sEMG amplitude (lower fatigue) observed in the examined muscles after therapy, may indicate that during contraction, the muscles activate less motor units to perform the same task compared to baseline.

The beneficial effect of neck-oriented therapy was presented in our study. Maluf et al. [51] also observed a decrease in pain intensity and tension of the neck and craniofacial muscles among patients with TMDs after stretching of the superficial neck and masticatory muscles. This effect may be explained by the fact that the muscles of the masticatory system are biomechanically, synergistic or antagonistic for the neck muscles, which means that they can support these muscles as cervical spine flexors or extensors. Therefore, correct functioning of the entire cervical muscular system is also crucial for healthy functioning of the masticatory system. Silveira et al. [52] evaluated the correlation among neck disability and TMJ dysfunction in subjects with TMD and concluded that high levels of neck disability correlated with high levels of jaw disability. They underlined the importance of considering the neck structures when evaluating and treating patients with TMD.

This study has some limitations which should be addressed. Due to the fact that we did not perform follow-up assessment after a longer period following the completion of therapy, we do not know how long the therapy effects last. Therefore, a study with a long time follow-up period and covering patients with dysfunctions different that idiopathic neck pain, is needed. Also because of ethical reasons we could not leave patients with neck pain without rehabilitation, therefore the control group included the healthy age-matched subjects without idiopathic neck-pain. So we can consider them only as a reference.

## Conclusions

In our research, it has been suggested that patients with idiopathic neck pain but without pain in the TMJ demonstrated neck and craniofacial muscle hyperactivity and impaired muscle coactivation, but after the therapy reached the values similar to that noted in the control group at baseline. So, we may consider values in the control group as a point of reference during the therapy in subjects with idiopathic neck pain. Three weeks of rehabilitation allowed to decrease the cervical and masticatory system muscles bioelectrical activity during rest as well as during movement and the level of those muscles’ fatigue. In this study, it has been demonstrated that therapy focusing only on the cervical region may improve the clinical condition of the masticatory system in subjects with idiopathic neck pain but without pain in the TMJ. It should be underlined that patients with neck pain, but without pain in the TMJ may, suffer from asymptomatic TMD. We have suggested that complex TMJ evaluation should always be included in the physiotherapy of patients with idiopathic neck pain, even if they do not report orofacial pain. In this study, it has been shown how close the functional relationship between neck and temporomandibular joints is, and thus, how dysfunctions in these areas affect each other. Thus, more research is needed to explore the implications of the treatment focused on cervical spine on TMD. These observations could be also helpful in the physiotherapeutic treatment of neck and craniofacial area dysfunctions.

## Supporting information

S1 File (DOC)

S2 File (PDF)

S3 File (PDF)

S4 File (PDF)
