# Correction: The influence of cervical spine rehabilitation on bioelectrical activity (sEMG) of cervical and masticatory system muscles

**DOI:** 10.1371/journal.pone.0271936

**Published:** 2022-07-18

**Authors:** Renata Kielnar, Anna Mika, Dorota Bylina, Jarosław Sołtan, Artur Stolarczyk, Błażej Pruszczyński, Henryk Racheniuk, Jan Szczegielniak, Aleksandra Królikowska, Łukasz Oleksy

The ninth author’s name is spelled incorrectly. The correct name is: Aleksandra Królikowska.
