# Supplementary material for: The influence of cervical spine rehabilitation on bioelectrical activity (sEMG) of cervical and masticatory system muscles
Source: PLoS One. 2021 Apr 26;16(4):e0250746. doi: 10.1371/journal.pone.0250746 (PMC8075221; doi:10.1371/journal.pone.0250746)
Supplement: S2 File — (PDF) [file pone.0250746.s002.pdf]

Komisja Bioetyczna Uniwersytetu Rzeszowskiego;
al. Tadeusza Rejtana 16C, 35-310 Rzeszów, Polska;
+48 17 872 19 20; komisjabioetur@gmail.com

Ocena funkcji mięśni stawów skroniowo-żuchwowych, mięśni szyi oraz mięśni przykręgosłupowych szyjnego odcinka kręgosłupa przed rehabilitacją i po jej zakończeniu u osób z idiopatycznym bólem w odcinku szyjnym

Cel badań

Ból szyi jest często spotykanym problemem, który objawia się u 70% osób w ciągu ich całego życia. Większość przypadków bólów szyi jest nieznanego pochodzenia, określane zwykle jako niespecyficzne lub idiopatyczne i niestety często nawracające. Piśmiennictwo światowe prezentuje wielomodułowe modele postępowania z pacjentem włączając w to ćwiczenia, aktywność fizyczną oraz terapię manualną. Jednym z sąsiadujących systemów szyjnego kręgosłupa jest narząd żucia. Kręgosłup szyjny jest ściśle z nim związany poprzez połączenia mięśniowe, nerwowe oraz poprzez ich wzajemną równowagę posturalną. Wszystko to ma fundamentalne znaczenie dla prawidłowego funkcjonowania tych struktur. Skupianie się w ocenie i terapii tylko na szyi, nie zawsze da nam oczekiwaną odpowiedź. W przypadku obecności zaburzeń w ich strukturach, dochodzić może do rozchwiania tej stabilizacji i pojawienia się zaburzeń świadczących o istniejących nieprawidłowościach. Współwystępowanie dolegliwości stawu skroniowo-żuchowowego wraz z zaburzeniami w obrębie kręgosłupa szyjnego jest dosyć powszechne. Coraz więcej klinicystów dostrzega u swoich pacjentów z dssż obecność symptomów i oznak zaburzeń odcinka szyjnego kręgosłupa, jednakże dokładny związek pomiędzy zaburzeniami szyi, a dssż, wciąż do końca nie jest jasny. To powoduje, że zazwyczaj terapia skupia się na leczeniu albo zaburzeń w stawie skroniowo-żuchwowym, lub dolegliwości z kręgosłupa szyjnego bez kompleksowego podejścia do problemu.

Celem badań jest ocena wpływu rehabilitacji szyjnego odcinka kręgosłupa na zakres ruchu w kręgosłupie szyjnym i stawach skroniowo-żuchwowych, na codzienną aktywność życiową oraz na aktywność mięśni przykręgosłupowych odcinka szyjnego kręgosłupa, mięśni narządu życia oraz mięśni szyi u pacjentów z idiopatycznym bólem szyi.

Materiał i metoda

Planowane jest objęcie badaniami 100 osób. Połowę (n=50) ma stanowić grupa osób z idiopartcznym bólem szyi, a drugą połowę (n=50) referencyja grupa zdrowych równolatków, bez dolegliwości ze strony kręgosłupa i stawów skroniowo-żuchwowych oraz nie będących w trakcie leczenia ortodontycznego.

Kryteria wyłączenia:

- Świeży uraz w odcinku szyjnym (do 3 miesięcy)
- Regularne stosowanie leków przeciwbólowych i sterydowych bez możliwości odstawienia ich podczas procesu rehabilitacji
- Zmiany w RTG odcinka szyjnego kręgosłupa (anomalia rozwojowe, obecność zmian degeneracyjnych, stenoza kanału kręgowego, zwyrodnienia kręgów szyjnych, podwichnięcia)
- Obecność przewlekłych chorób współistniejących
- Leczenie ortodontyczne (szyny nocne, aparaty ortodontyczne), proteza zębowa ruchoma

Pacjenci z idiopatycznym bólem szyi będą poddani 3 tygodniowej kompleksowej rehabilitacji szyjnego odcinka kręgosłupa.

Program rehabilitacji zawierał będzie następujące elementy:

- Edukacja pacjenta dotycząca przyczyn dysfunkcji, technik korekcji postawy ciała i erogonomii w codziennym życiu.
- Terapia tkanek miękkich okolicy odcinka szyjnego kręgosłupa i obręczy barkowej (terapia punktów spustowych, rozluźnienie mięśniowo-powięziowe, poizometryczna relaksacja mięśni)
- Manualna trakcja szynego odcinka kręgosłupa
- Fizykoterapia (lampa sollux)
- Masaż klasyczny
- Indywidualne ćwiczenia z terapeutą

Na początku i po 3 tygodniach osoby te będą poddane następującym badaniom:

- Ocena intensywności bólu za pomocą skali VAS
- Badanie fizykalne odcinka szyjnego kręgosłupa: ocena ruchomości odcinka szyjnego kręgosłupa charakterystyka fizjologicznych krzywizn kręgosłupa, ustawienia głowy (postawa ciała) - za pomoca taśmy centymetrowej

- Badanie kliniczne i ocena stawów skroniowo – żuchwowych przy użyciu wskaźnika Helkimo (Di).
- Ocena aktywności bioelektrycznej mięśni sEMG (mm. mostkowo-obojczykowo-sutkowe, mm. przykręgosłupowe szyi (okolica C4), mm. czworoboczne - część zstępująca, mm. żwacze, mm. skroniowe (część przednia). Pomiary będą wykonane podczas siedzenia w pozycji swobodnej i skorygowanej, podczas wybranych czynnych ruchów w szyjnym odcinku kręgosłupa oraz zaciskania zębów.
