# Supplementary material for: The influence of cervical spine rehabilitation on bioelectrical activity (sEMG) of cervical and masticatory system muscles
Source: PLoS One. 2021 Apr 26;16(4):e0250746. doi: 10.1371/journal.pone.0250746 (PMC8075221; doi:10.1371/journal.pone.0250746)
Supplement: S3 File — (PDF) [file pone.0250746.s003.pdf]

Ethical Committee of Rzeszow University in Poland;
al. Tadeusza Rejtana 16C, 35-310 Rzeszów, Poland;
+48 17 872 19 20; komisjabioetur@gmail.com

Impact of cervical spine rehabilitation on temporomandibular joints functioning, and bioelectrical activity of cervical and masticatory system muscles in patients with idiopathic neck pain

Background and study aim

Cervical spine dysfunction is a cause of neck pain. Cervical spine dysfunction occurs in about 70% of the population. Both diagnosis and treatment of these problems are difficult, and in many cases, the effects of therapy are not satisfactory. Existence of jaw (temporomandibular joint) discomfort along with cervical spine disorders is quite common and is associated with many limitations and adverse symptoms for the patient. The exact relationship between neck ailments and temporomandibular dysfunction (TMD) is still unclear. This study aims to verify the functional relationship between the cervical spine and TMJ as well as check whether the therapy focusing only on the cervical region has a real impact on the structures located in the facial area. These tests have practical applications because they can indicate new directions in the diagnosis and therapy of neck and TMD

Methods

The study will recruit 100 participants. Experimental group will include 50 patients with idiopathic neck pain. Experimental group will recieve three-weeks rehabilitation program, individual for each patient and comprise the following standard treatments for chronic pain of the musculoskeletal system.

Participant exclusion criteria

1. Cervical spine injury 3 months prior to the therapy
2. Regular use of painkillers or steroids, without possibility to withdraw it for the whole duration of the therapy
3. Radiographically diagnosed developmental and degenerative abnormalities of the cervical spine as; spinal stenosis, subluxations, spinal disc herniation
4. Orthodontic treatment (braces, aligners); removable dentures

Experimental group will recieve three-weeks rehabilitation program, which comprises the following standard treatments for chronic pain of the musculoskeletal system: manual therapy (soft tissue therapy of the neck and the shoulder girdle, trigger point therapy, manual cervical traction, classical massage, myofascial release); individual exercises with a therapist (weight free, respiratory, fit ball and body posture correction exercises); physical therapy (sollux lamp); education on the character of the dysfunction, body posture correction techniques and ergonomics.The rehabilitation will be provided five times a week lasting each time for about two hours.

Age-matched subjects (control group; n=50) who will be cervical pain-free, have no cervical spine and TMJ dysfunctions or are not in the process of current orthodontic treatment will not recieve any therapy.

Primary outcome measure will be assessed at baseline and after 3 weeks:

1. Pain intensity measured using 10-point visual analogue scale (VAS)
2. Temporomandibular joints functioning measured using the Helkimo clinical dysfunction index (Di)
3. Head posture and range of motion in the cervical spine - measured with measuring tape
4.Cervical Spine and craniofacial muscles bioelectrical activity (sEMG) assessed by surface electromyography
